# Potential contribution of gut microbiota and systemic inflammation on HIV vaccine effectiveness and vaccine design

**DOI:** 10.1186/s12981-017-0164-9

**Published:** 2017-09-12

**Authors:** Jean-Pierre Routy, Vikram Mehraj

**Affiliations:** 1 Research Institute of the McGill University Health Centre, Montreal, QC Canada; 2 Chronic Viral Illness Service, McGill University Health Centre, Montreal, QC Canada; 3 Division of Hematology, McGill University Health Centre: Glen Site, Research Institute, Block E, Suite EM 3-3232, Mezzanine 3M, 1001 Boulevard Décarie, Montreal, QC H4A 3J1 Canada

**Keywords:** HIV vaccine, Immune activation, Inflammation, Microbial translocation, Correlate and surrogate of vaccine protection, Gut microbiota

## Abstract

The quest for an effective HIV-1 vaccine began as soon as the virus causing AIDS was identified. After several disappointing attempts, results of the Phase-III RV144 trial in Thailand were a beacon of hope for the field demonstrating correlation between protection and immunological markers. In order to optimize vaccine response, we underline results from yellow fever and hepatitis B vaccines, where protective responses were predicted by the pre-vaccination level of immune activation in healthy individuals. Such findings support the assessment and reduction of pre-vaccine immune activation in order to optimize vaccine response. Immune activation in healthy individuals can be influenced by age, presence of CMV infection, gut dysbiosis and microbial translocation. We speculate that the level of immune activation should therefore be assessed to better select participants in vaccine trials, and interventions to reduce inflammation should be used to increase protective HIV vaccine response.

## Background

Despite tremendous effort to develop a successful human immunodeficiency virus (HIV) vaccine, the quest for a safe and effective HIV vaccine seems to be remarkably long and winding. Most licensed vaccines against viral and bacterial infections demonstrate efficacy in inducing antibodies [1]. In addition to the induction of specific antibodies, vaccines also induce protective immunity by eliciting a combination of innate and acquired cellular responses [2]. However, specific immune responses following administration of licensed vaccines is not well-understood, but have recently been shown to be associated with pre-vaccine level of immune activation [3]. In contrast to the clearance of childhood viral infections like smallpox and measles, vaccine development against HIV remains challenging as the virus inexorably causes a chronic infection that cannot be naturally cleared by the host. Furthermore, there is little guidance in vaccine design when considering the determinants of host response, due to the absence of spontaneous clearance of HIV infection. Development of an HIV vaccine is even more difficult considering the unparalleled capacity of HIV to mutate its genome, allowing it to evade antibody recognition of its viral envelope [4], in addition to its ability to suppress the major conductor of the immune response, the helper CD4 T-cells [5]. The ideal HIV vaccine should provide protection against the broad genetic diversity of HIV, control CD4 T-cell activation and likely elicit long-term cytotoxic CD8 T-cell response to block both mucosal and parenteral transmission routes.

The humoral immune response is one of the first lines of defense against invading pathogens, which plays a crucial role in preventing infection. Vaccine efficacy can be predicted using a correlate of protection (CoP), which is a measurable immune biomarker that identifies immunity in the host. The antibody titre represents the most common CoP for licensed vaccines [6]. However, establishing immune determinants responsible for the protection remain difficult as several type of immune responses are intertwined [7]. Importantly, such immune responses may depend on type of vaccine, level of immune activation, micro-environment and host genetic factors. Very limited information exists on CoP in HIV as vaccine efficacy has been globally disappointing.

One of the most encouraging vaccine studies RV144, investigated the efficacy of a combined vaccine regimen in young adults in Thailand [8, 9]. The vaccination regimen consisted of ALVAC-HIV boosted with AIDSVAX^®^ B/E, and demonstrated partial efficacy of 31% against HIV-1 infection. The level of protection was associated with the development of envelope specific antibodies [9, 10]. In addition, antibody-dependent cell-mediated cytotoxicity (ADCC), host genetics and virus diversity in HIV-1 were also correlates of protection. Such correlates are defined as vaccine-induced immune responses associated with the rate of HIV-1 infection among vaccine recipients. Measuring these forms of CoP can be labor intensive, costly, and not scalable, highlighting the need to identify surrogate markers that can simply and reliably predict the level of protection from HIV-1 infection.

## Immune activation as newly identified correlate of protection: evidence from yellow fever and hepatitis B commercialized vaccines

Recent information generated from yellow fever and hepatitis B on CoP will be useful to further guide HIV vaccine development and implementation.

### Yellow fever vaccine

In a 2014 study, Muyanja and colleagues sought to understand the modulation of vaccine immune responses towards better guidance in vaccine development against difficult targets like HIV or malaria [11]. As such, they evaluated patient-specific immune responses to the commercialized yellow fever vaccine, YF-17D. By comparing healthy individuals from Lausanne (Switzerland) and Entebbe (Uganda), the authors showed that YF-17D induced B cell responses, as well as a substantial reduction in CD8 T-cells in age-matched individuals living in Entebbe [11]. Furthermore, the authors identified higher frequencies of exhausted and activated NK cells, more differentiated B and T-cell subsets, and an abundance of proinflammatory monocytes, demonstrating an activated immune microenvironment in the participants from Entebbe. Baseline B and CD8 T-cell activation levels, coupled with proinflammatory monocytes, negatively predicted titres of YF-17D-neutralizing antibody post vaccination. In addition, B cell and memory T-cell responses measured before vaccination in the Entebbe did not last as long as in the Lausanne participants. Such findings in healthy individuals indicate that immune activation prior to vaccination influences the response of one of the most protective vaccines ever commercialized, and illustrates the importance of considering immune modulation to optimize HIV vaccine response.

The importance of immune modulation in vaccine response is supported by a recent study that investigated the impact of infection history in a mouse model. Since laboratory-raised mice are not typically exposed to common infections acquired by human children, these mice may not be the best model to predict immune responses in humans. Blood immune responses were measured before and after YFV-17D vaccination in mice that were sequentially infected with herpes viruses, influenza and a helminth [12]. The authors uncovered significant differences in gene expression patterns between mock and co-infected samples, both before and after vaccination. They also found that co-infected mice had a reduced antibody response, but had similar neutralizing antibody titers after vaccination. As a consequence of this work, ongoing clinical trials are assessing the effect of an anthelminthic treatment before yellow fever vaccination to enhance protective vaccine response.

### Hepatitis B vaccine

To further elucidate the factors influencing CoP, the Sekaly group studied vaccine response in elderly participants following hepatitis B vaccination [3]. After measuring gene expression profiles according to age, they assessed pre-vaccination predictors of response to HBV vaccine in distinct sets of older adults. A stronger HBV response was observed for those having heightened expression of genes that augment B-cell responses and higher memory B-cells. Conversely, higher levels of inflammatory response transcripts and increased frequencies of pro-inflammatory innate cells were associated with a lower vaccine response. Again, this work identified the contribution of immune activation as a key baseline determinant of HBV vaccine response, which may also be relevant for future HIV vaccine development.

Other host and environmental factors that may influence CoP are also intertwined with immune activation. Host factors include immune exhaustion, senescence, metabolic reprogramming and tissue inflammation, contributing to T-cell dysfunction and modulation of B-cell response to neo-antigens or vaccines. Among the environmental factors, gut microbial composition, gut mucosal barrier damage and cytomegalovirus (CMV) co-infection are the most studied and differ in Northern and Southern hemispheres [13].

## Relevance of immune activation to improve HIV vaccine response

As noted, the encouraging results of the Thai RV144 vaccine trial provided insight into future HIV vaccine development [9]. The investigators identified that IgG for the V1V2 region of Env gp120 was associated with protection from infection. However, the protection did not persist and was temporally linked with antibodies mediating antibody-dependent-cellular cytotoxicity (ADCC). Importantly, mounting evidence indicates that antibody mechanisms beyond neutralization may contribute to protection, as Fc characteristics and ADCC have been identified as CoP against HIV acquisition conversely to the induction of neutralizing antibodies and/or cytotoxic T-cells [8]. Such findings suggest that combinations of broad antibodies targeting different binding sites on the Env protein could block immune escape. More recent findings from this trial support correlation between T follicular help (Tfh) function relevant for B-cell help and envelope-specific antibody development. Tfh responses generated by RV144 show a marked inter-individual variation indicating the presence of determinants that remain largely unknown [14].

Observations of inter-individual variation highlight a knowledge gap in the role of human pre-immune repertoires in driving vaccine response. One such repertoire is the gut microbiota, as it can elicit influences that are indirect and can act at a distance. These mechanisms may involve cross-reactivity between microbial and vaccine antigens shaping T-cell repertoires, and/or microbial products stimulating pattern recognition receptors that influence the type and intensity of vaccine responses. Preliminary data show that T helper cells cross react with HIV antigens which are identified in uninfected healthy individuals and are usually missed by standard intracellular assay for the measurement of Th1 cytokines [15]. Such cross-reactive responses may shape subsequent responses to HIV vaccine candidates in both magnitude and function [16]. This is further illustrated by the HVTN 055 trial which showed a lack of protection against HIV, owing to the unexpected induction of non-neutralizing gp41-reactive anti-HIV antibodies [17]. The antibodies elicited by the vaccine were related to a polyreactive antibody repertoire response from pre-existing B-cells that were cross-reactive with *Escherichia coli* likely originating from the gut. Understanding of the contribution of the microbiome and/or microbial translocation to both vaccine-induced immunity and non-specific immune activation represents a new and unforeseen challenge for the development of an optimal vaccine response.

**Fig. 1:**
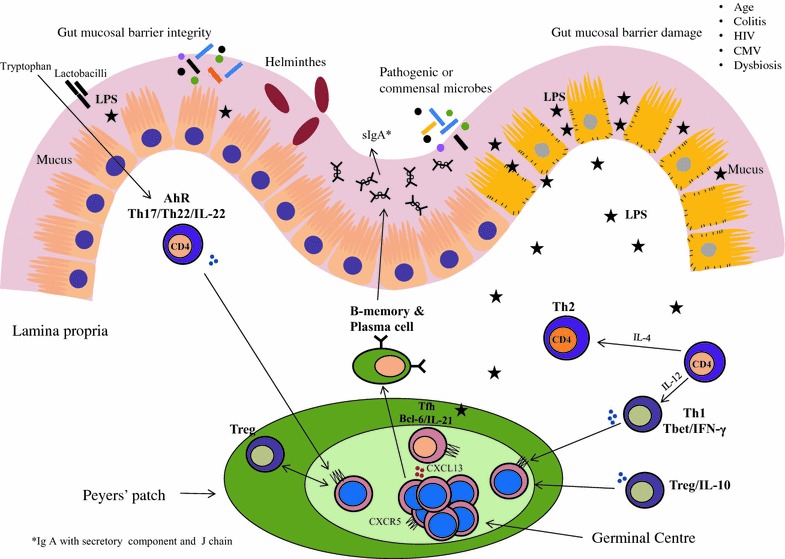
Contribution of gut microbes and microbial translocation on immune response pertaining to HIV vaccine development

Gut-associated lymphoid tissue (GALT) represents a major site of immune response [18, 19]. This is due to the GALT that harbors a large fraction of activated CD4 T-cell populations, which are preferential targets for HIV replication. The interaction of GALT CD4-T cells with commensal/pathogenic microflora promotes cellular activation that favors immune activation. In the context of treated HIV-infection, the predictors of commercialized vaccine response are linked with the levels of T-cell activation and translocation of bacterial products such as LPS into the systemic circulation [20] (Fig. 1). LPS-triggered toll-like receptor (TLR) signaling induces development and activation in the B-cells in the germinal centres (GCs) thereby regulating GC formation and antibody production [21]. Such immune activation has also been characterized by the induction of B-cell attracting chemokine, CXCL13, which is recognized by the receptor CXCR5, and contributes to the follicular homing of B and T-cells. Unlike most healthy tissue, the normal intestinal lamina propria contains a large number of plasma cells containing IgA. Secretary IgA (sIgA) is almost completely dependent on the presence of microbiota which predominate the distal colon [22], which may influence the HIV vaccine specific IgA response [23] (Fig. 1).

## Gut microbiota, metabolites and vaccine response

Manipulation of microbiota composition and their metabolites via diet alteration or microbiota engraftment is under intense evaluation in cancer and autoimmune disorders and vaccinology. Learning from our gut endogenous original adjuvants and/or tolerogenic microbes and their metabolites will be critical in overcoming the HIV vaccine challenge [24]. We recently showed the contribution of dietary tryptophan (Trp), one of the essential amino acids mainly obtained from protein-rich foods, contributed to immune suppression by the production of an immunosuppressive catabolite in the context of HIV/HCV infections [25–27]. Other groups have identified a similar change in cancer, autism, and multiple sclerosis indicating the influence of kynurenine, a metabolite of Trp used in the production of niacin, as an immunosuppressor present in several chronic conditions [28, 29]. Of note, several Trp metabolites produced by bacteria in the gut are endogenous ligands for the transcription factor aryl hydrocarbon receptor (AhR) which has been recently shown to control B-cell fate decisions including suppression of class switching in vivo after influenza immunization [30]. AhR activation is linked to both diet and gut microbiota composition and leads to the local production of IL-22, a cytokine that plays an important role in maintaining mucosal immunity and integrity, mainly by innate lymphoid cells group 3 (ILC3) [31, 32]. Only specific subsets of bacteria, particularly Lactobacilli, an important member of Firmicutes, can metabolize dietary Trp into idole-3-aldehyde to modulate AhR/IL-22 axis, thus contributing to gut mucosal homeostasis [32, 33]. In addition, AhR activation occurring in macrophages and DCs further contributes to the local anti-inflammatory response.

Certain microbes like *Taenia crassiceps*, a parasite that causes a gut helminthic infection, have been shown to impair antibody response to pneumococcal vaccine in mice [34]. Similarly, Riner et al. showed that immunization against hepatitis B and tetanus toxoid in Kenyan adults having schistosomiasis, caused by members of the Schistosoma genus, resulted in a more rapid decline in antibody titres that can be prevented by a prior anti-helminthic treatment [35]. All these observations highlight the importance of gut microbial dysbiosis on distal immune response in physiology and disease [36].

## CMV infection, aging and vaccine response

Human cytomegalovirus (CMV) establishes a latent infection that remains generally asymptomatic but can lead to serious illness in immune-suppressed individuals [37, 38]. The long-term control of CMV shapes the immune system contributing to a memory inflammation induced by CD8 T-cell specific response. Such CMV-associated low-grade inflammation also contributes to human aging and has been termed as “inflammaging” [39, 40]. CMV seropositivity has been shown to have a negative effect on influenza vaccine-specific antibody responses in the elderly as well as in the young [41]. CMV infection is prevalent in Africa and may contribute to a lower vaccine response observed in this population. Furthermore, CMV co-infection has been associated with faster disease progression and elevated CMV-specific IgG antibody levels in untreated HIV-infected individuals, further contributing to disease progression and immune activation [42]. For patients receiving long-term antiretroviral therapy (ART), CMV-co-infection remains an independent contributor to persistent CD8 T-Cell expansion and inflammation [43].

## Conclusions

We are starting to appreciate the importance of pre-existing inflammation linked to gut microbial composition in vaccine response that should influence HIV vaccine development where the bulk of epidemic prevails in developing countries. The complex interplay between microbial composition and the gut epithelial barrier contributes to the state of systemic immune activation representing an emerging area to be considered to optimize HIV vaccine response. Modulation of chronic immune activation prior to vaccine administration may elicit persistent memory antibody response with CD8 T-cell cytotoxic function. Collaborative effort between microbiologists, immunologists epidemiologists and clinicians will be needed to foster HIV vaccine research.
